# Left main occlusion – a classic electrocardiogram 

**DOI:** 10.1007/s00508-016-1007-8

**Published:** 2016-06-08

**Authors:** Johannes Peter Schwaiger, Johannes Mair

**Affiliations:** Department of Internal Medicine, Landeskrankenhaus Hall, Hall, Austria; Department of Internal Medicine III – Cardiology and Angiology, Medical University Innsbruck, Innsbruck, Austria

**Keywords:** Myocardial infarction, Cardiac catheterization, Coronary angiography, Echocardiography, Ventricle

## Abstract

Left main occlusion is associated with a dire prognosis, as the left main supplies blood to >75 % of the left ventricle. This is a case showing a classic electrocardiogram (ECG) together with the echocardiogram videos and images from coronary angiography.

**Video online:** The online version of this article (doi: 10.1007/s00508-016-1007-8) contains supplementary material, which is available to authorized users.

An 83-year-old male was admitted to hospital because of mild chest pain lasting for 2 hours. His past medical history included paroxysmal atrial fibrillation, hypertension, and a history of prostate carcinoma, for which he had undergone prostatectomy in the past. His medication included a vitamin K antagonist, amiodarone, enalapril, amlodipine, hydrochlorothiazide*, *casodex, and tamoxifen.

On examination, the patient’s heart rate was 53 bpm (regular), blood pressure 126/83 mmHg, 96 % oxygen saturation in room air. Auscultation of heart and lung was unremarkable; there were no clinical signs of heart failure.

The ECG (Fig. 1) showed sinus rhythm, complete right bundle branch block, left anterior hemiblock, and atrioventricular (AV) block type I; as well as pronounced descending ST segment depression in the inferior and lateral leads, but also ST segment elevation of about 0.1 mV in lead V1 and approaching 0.2 mV in lead aVR.

**Fig. 1:**
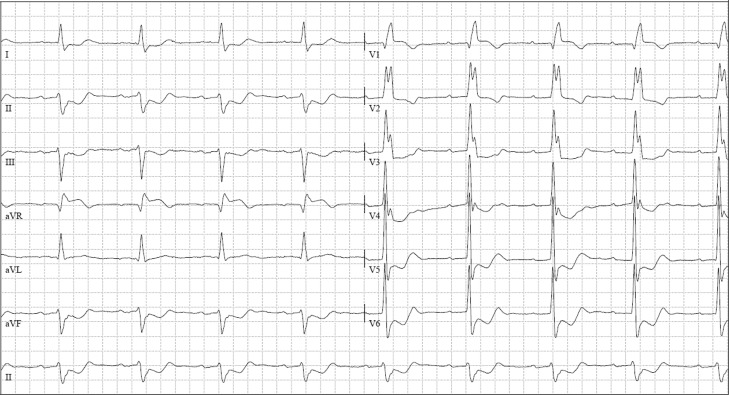
ECG on admission: sinus rhythm, complete right bundle branch block, left anterior hemiblock, AV block type I, descending ST segment depression in inferior and lateral leads, ST segment elevation of about 0.1 mV in lead V1 and approaching 0.2 mV in lead aVR

Blood results showed a high-sensitivity troponin T (hs-cTnT) value of 19 ng/l, haemoglobin 11.6 g/l, creatinine 1.3 mg/dl, a subtherapeutic international normalized ratio (INR) of 1.3, and were otherwise unremarkable.

A presumed diagnosis of non-ST-elevation myocardial infarction was made and the patient transferred to a monitored unit. He received nitroglycerin sublingually, aspirin, clopidogrel, and one dose of enoxaparin. His chest pain improved somewhat but did not completely subside.

Echocardiography showed severely reduced global left ventricular ejection fraction, with akinesia of anterior and severe hypokinesia of inferolateral walls (videos 1–3). A repeat blood test 2 h after the first test revealed an increase in hs-cTnT to 46 ng/l. ST segment changes had improved but were still present, as was the chest pain. The patient was then transferred to the local primary percutaneous coronary intervention (PCI) center for cardiac catheterization.

Coronary angiography showed a complete occlusion of the left main coronary artery (Fig. 2), as well as a dominant right coronary artery delivering collaterals to a chronically occluded left anterior descending (LAD) artery and diagonal territory without significant stenosis (Fig. 3). Primary PCI with left main stenting was performed and could restore blood flow to the circumflex artery. An additional chronic total occlusion of left anterior descending artery in its mid region could not be reopened during the procedure.

**Fig. 2:**
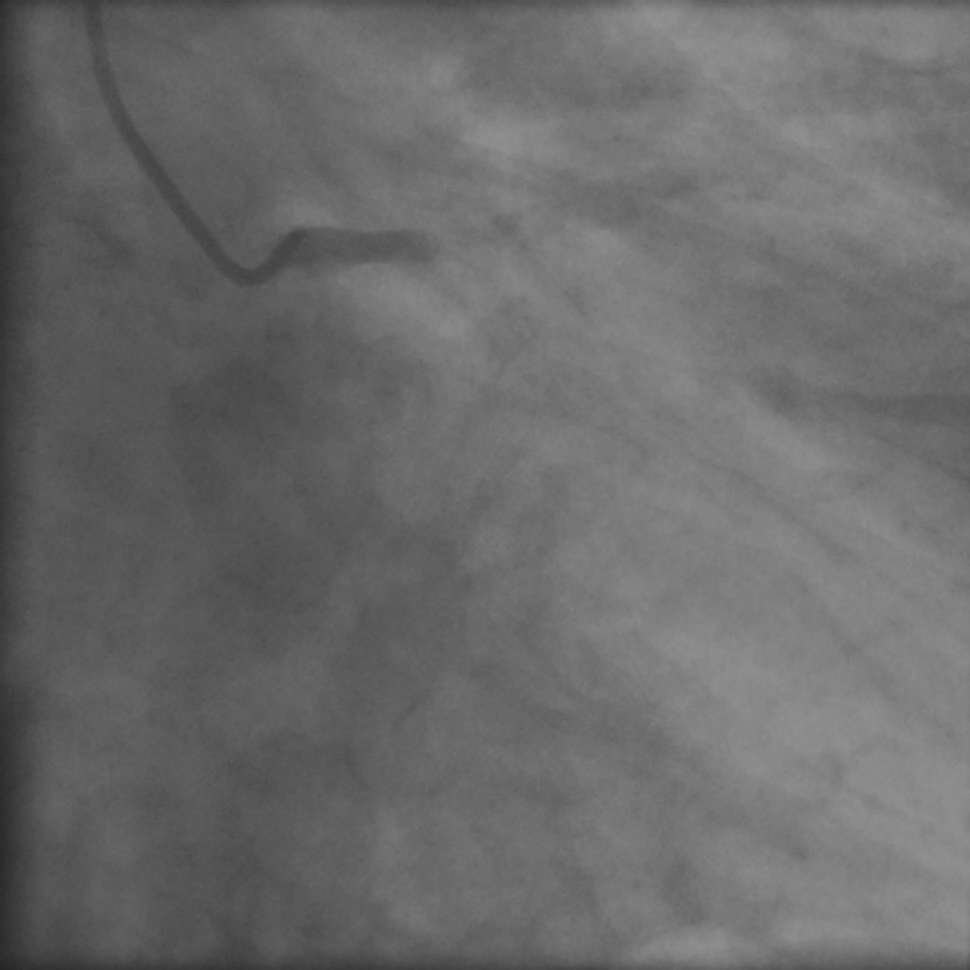
Complete occlusion of the left main coronary artery

**Fig. 3:**
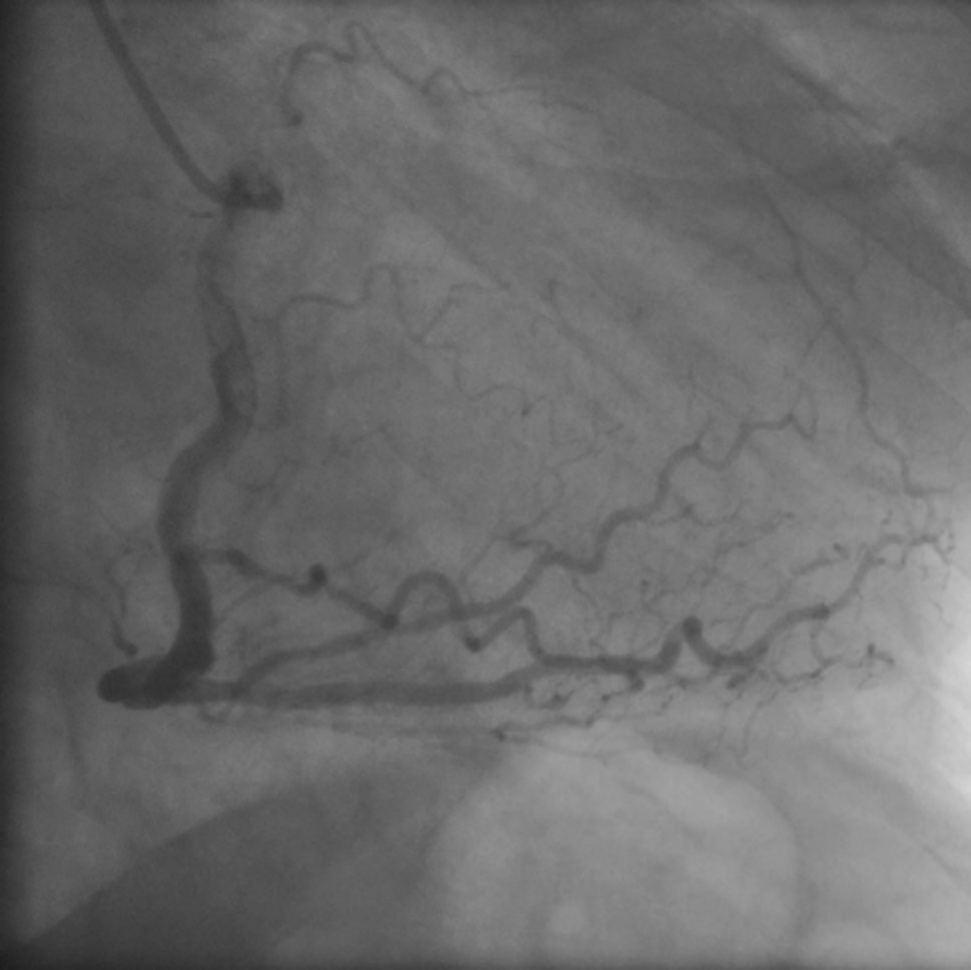
Dominant right coronary artery delivering collaterals to a chronically occluded left anterior descending artery and diagonal territory without significant stenosis

## Conclusion

Left main occlusion is associated with a dire prognosis, as the left main supplies blood to >75 % of the left ventricle. Mortality is high and is usually dependent on preexisting collaterals from the right coronary system, as in our patient. ECG changes were typical and included diffuse ST segment depression in inferior and lateral leads, but also ST segment elevation in aVR and less in V1. It is worth noting that ST segment elevation in aVR is reciprocal to ST depression and not ST elevation per se, and can also occur in proximal LAD occlusion.

## Caption Electronic Supplementary Material







